# Perceived Benefits and Harms of the COVID-19 Pandemic on Family Well-Being and Their Sociodemographic Disparities in Hong Kong: A Cross-Sectional Study

**DOI:** 10.3390/ijerph18031217

**Published:** 2021-01-29

**Authors:** Bonny Yee-Man Wong, Tai-Hing Lam, Agnes Yuen-Kwan Lai, Man Ping Wang, Sai-Yin Ho

**Affiliations:** 1 School of Public Health, The University of Hong Kong, Hong Kong, China; bonnyyw@hku.hk (B.Y.-M.W.); hrmrlth@hku.hk (T.-H.L.); 2 School of Nursing, The University of Hong Kong, Hong Kong, China; agneslai@hku.hk

**Keywords:** COVID-19, perceived harms, perceived benefits, family well-being, sociodemographic disparities

## Abstract

We assessed the perceived benefits and harms of COVID-19 on family and their associations with sociodemographic factors in Chinese adults in Hong Kong. We conducted an online population-based survey and collected 4891 responses in 6 days. Prevalence estimates were weighted by sex, age, and education of the general population, and associations were analyzed using logistic regression. Our results showed both perceived benefits: 19.0% for family physical health, 7.2% family mental health, and 13.5% family relationships; and harms: 2.3% for family physical health, 37.9% family mental health, 18.6% family relationships, and 37.8% decreased family income. More female or older respondents reported perceived benefits but fewer of them reported perceived harms. More respondents with higher than lower socioeconomic scores (SES) reported perceived benefits on family physical and mental health and family relationships, but more respondents with lower than higher SES reported perceived harm on family income. As the pandemic continues with uncertainties, further studies on the dynamics of benefits and harms are needed. Urgent and additional assistance to underprivileged families and at-risk individuals are needed to reduce the inequities amidst the COVID-19 pandemic.

## 1. Introduction

The COVID-19 pandemic has spread rapidly since early 2020 [1]. The World Health Organization published a Strategic Preparedness and Response Plan to support country preparedness and responses at regional, national, and subnational levels [2]. Various social distancing measures, such as border restrictions, closures of schools and nonessential businesses, and stay-at-home orders were enforced in many countries in response [3].

The Hong Kong Special Administrative Region is the most developed and crowded city in China, with over 7 million people, and is closely connected with mainland China, including the epicenter of Wuhan. From 23 January to 31 May 2020 (around the end of Wave 2), 1084 confirmed COVID-19 cases were reported [4]. Hong Kong responded quickly with voluntary mask wearing and hand hygiene in the early stage of the pandemic [5]. Solidarity and altruism were advocated early in April 2020 by one of the co-authors (THL) of the present paper, among others [6]. Both Waves 1 (imported cases from mainland China with limited local cases) and 2 (imported cases from other countries leading to more local cases) were controlled by nearly 100% voluntary masking, hand hygiene, and government public health measures (isolation, quarantine, contact tracing, and social distancing) without lockdown [6,7].

In addition to the massive and increasing number of confirmed cases and deaths, the COVID-19 pandemic causes many other harms on individuals and families, such as social isolation, distress, anxiety, and depression [8,9,10,11,12,13,14,15]. The pandemic also increased unpaid household work (including child care) time, and decreased paid job time [16,17,18], hence financial loss [18]. 

However, the pandemic could have some potential benefits, such as increased family time and hygiene. We used keywords including “COVID-19”, “SARS CoV 2”, “Severe Acute Respiratory Syndrome Coronavirus 2”, “2019 nCoV”, “family”, “relationship”, “well-being”, “communication”, “disparity”, and “inequality” to search PubMed and Web of Science up to 1 October 2020 and found no survey reports on both benefits and harms of COVID-19 on the same individuals and families. Only one survey in Spain asked an open-ended question: “What changes have you perceived in your couple or family dynamics since the beginning of home confinement?” [19]. Qualitative analysis of this question identified perceived deterioration and perceived improvement of family relationships during the lockdown as main themes. Deterioration themes included loneliness, couple/family distance, and the conflict atmosphere; improvement themes included family (re)connection and acknowledgment, better communication, and emotional expressiveness [19].

Five studies reported sex, age, and education differences on mental health, work arrangement, and income during the COVID-19 pandemic [11,13,18,20,21]. More women reported feelings of depression and anxiety [20] and younger adults reported poorer mental health during the COVID-19 pandemic [11,13]. Younger workers and those with less education or less income were more likely to lose income and/or jobs and less likely to be able to work from home during the pandemic than their respective counterparts [18,21].

Under the Hong Kong Jockey Club Smart Family-Link Project, we conducted the Family Amidst COVID-19 (FamCov) survey after the second wave was under control. Using data from FamCov, this paper aims to describe the perceived benefits and harms of COVID-19 on family physical, mental, relational, and financial well-being in Hong Kong Chinese adults and to analyze the sociodemographic disparities of such benefits and harms.

## 2. Materials and Methods 

### 2.1. Sample and Procedures

Because we aimed to measure the point prevalence of many previously unknown factors and outcomes and their associations (if any), our strategy was to recruit as many respondents as practicable with the budget constraint and in a short period of time (i.e., less than one week) before the next wave of the outbreak by inviting both probability- and non-probability-based online panels through the Hong Kong Public Opinion Research Institute, a well-known survey agency locally. The probability-based panel included persons who were randomly selected in previous telephone surveys and representative of the Hong Kong population, whereas the non-probability-based panel included any person in Hong Kong who volunteered to join through completing an online form [22]. Because there were no validated questionnaires on perceived harms and benefits from COVID-19, we designed the questions and did some pilot tests. Respondents showed no difficulties in responding, suggesting face validity. The online survey was sent to 70,984 adults aged 18 years or above with valid email addresses from the panels.

However, the invitation email might have been automatically classified as spam and trashed, hence only 20,103 invitation emails were opened and 6956 accessed the survey link within 6 days (from 26 to 31 May 2020). Of these, 4921 provided useable data after giving informed consent (response rate = 4921/20,103 = 24.5%). As the study objectives were about families, we excluded 30 respondents without family members, leaving 4891 for the analyses. With the assumption of a 2% margin of error, 99% confidence interval, 50% of population, and 6,500,000 persons aged 18 years or above in mid-2019 in Hong Kong [23], the estimated sample size was 4145, using

$$Sample\;size=\frac{\frac{z^{2}\times p\left(1-p\right)}{e^{2}}}{1+\left(\frac{z^{2}\times p\left(1-p\right)}{e^{2}N}\right)}$$

where *N* = population size, *e* = margin of error, *z* = z-score (corresponding to confidence interval), *p* = proportion of population with the attribute [24]. Ethics approval was granted by the Institutional Review Board of the University of Hong Kong/Hospital Authority Hong Kong West Cluster (UW 20-238).

### 2.2. Measures

#### 2.2.1. Dependent Variables

Perceived benefits of COVID-19 were assessed by the question “What benefits have COVID-19 outbreak brought to your family (including yourself)?” The 7 options of benefits included in the present paper were grouped into 3 areas: (1) family physical health (improved family hygiene and improved family physical health); (2) family mental health (decreased family negative emotion, increased family positive emotion, and increased family happiness); and (3) family relationship (increased family harmony and increased family’s ability to cope with difficulties). Each perceived benefit was analyzed as yes vs. no.

Perceived harms of COVID-19 were assessed by the question “What harms have COVID-19 outbreak brought to your family (including yourself)?” The 7 options of harms were grouped into 4 areas: (1) poorer family physical health; (2) family mental health (increased family negative emotion and decreased family happiness); (3) family relationship (decreased family harmony, decreased family’s ability to cope with difficulties, and increased family conflicts); and (4) decreased family income. Each perceived harm was analyzed as yes vs. no.

To assess the validity of responses, counterfactual options of perceived benefit of increased family income and perceived harm of poorer family hygiene that were unlikely to be selected were provided. Indeed, less than 0.01% (17 and 16 respondents, respectively) chose these options, providing support for the validity of data.

#### 2.2.2. Independent Variables

Information on sex, age group (18–24, 25–34, 35–44, 45–54, 65 years or above), education (primary or lower, secondary, diploma or certificate, associate degree, and degree or higher), household monthly income (no income, less than HKD 4000, HKD 4000–9999, HKD 10,000–19,999, HKD 20,000–29,999, HKD 30,000–39,999, and HKD 40,000 or higher (USD 1 = HKD 7.8), number of cohabitants, and housing (public housing, subsidized housing, private housing (rent), and private housing (owned)) was collected. Sociodemographic variables were recoded: age (18–24, 25–44, 45–54, and 65 years or above), education (secondary or below, and postsecondary), and housing (rent and owned). Household monthly income per person (income divided by household size) was derived and dichotomized with reference to official figures into “lower” (equal to or less than the household size specific median household monthly income of Hong Kong) or “higher” [25]. A socioeconomic score (SES) ranged 0–3 was created by summing scores from education (0 = secondary education or lower, 1 = postsecondary education), household monthly income per person (0 = lower, 1 = higher), and housing (0 = rent, 1 = owned). The SES was further coded into low (0–1), middle (2), and high (3) due to the similar characteristics of those with scores of 0 and 1. 

### 2.3. Statistical Methods

All statistical analyses were conducted in *IBM SPSS v. 27* and *p* < 0.05 was considered statistically significant. Descriptive statistics were used to describe the sociodemographic characteristics and perceived benefits and harms of COVID-19 with rim-weighting by sex, age, and education of the Hong Kong general population so that the results would be more representative [23,26]. 

Chi-squared tests were used to examine sociodemographic differences in perceived benefits and harms, and Mantel–Haenszel tests were used to test linear trends. Multivariate logistic regression yielded adjusted odds ratio (OR) and 95% confidence interval (CI) of perceived benefits and harms for sociodemographic variables with mutual adjustment. 

## 3. Results

### 3.1. Participants

Table 1 shows that in the weighted sample, the most common perceived benefits of COVID-19 on family well-being were improved family hygiene (17.8%), improved family physical health (10.9%), and increased family’s ability to cope with difficulties (10.6%). On the other hand, the most common perceived harms were decreased family income (37.8%), increased family negative emotion (33.3%), and decreased family happiness (18.9%). The prevalence of perceived benefits on family physical health, mental health, and relationships was 19.0%, 7.2%, and 13.5%, respectively. The corresponding figures for perceived harms were 2.3%, 37.9%, and 18.6%. These weighted prevalence values were very similar to the unweighted ones (with differences of a few percentage points).

### 3.2. Sex, Age, and SES Differences in Perceived Benefits and Harms 

Table 2 shows significant sex differences with more females than males reporting perceived benefits of COVID-19 on family mental health (9.0% vs. 7.0%, *p* = 0.012) and relationships (18.2% vs. 14.2%, *p* < 0.001), but more males than females reported perceived harms on family mental health (39.3% vs. 35.1%, *p* = 0.005). More respondents aged 65 years or above reported perceived benefits on family mental health (14.7%) and family relationships (23.1%) than the three younger age groups (ranging from: 6.7%–9.2% and 14.4%–18.3%, respectively) (all Ps < 0.01). More respondents aged 18–24 years reported perceived harms on family relationships (28.4%) than the three older age groups (9.6%–23.7%) (*p* < 0.001). The linear trends of these three outcomes were significant (all Ps < 0.001).

Table 3 shows that significantly more respondents with higher than lower SES reported perceived benefits on family physical health (24.4% vs. 17.5%) and family relationships (18.6% vs. 11.3%) (all Ps < 0.01). On the other hand, more respondents with low SES reported the perceived harm of decreased family income (41.0%) than those with middle (32.5%) and high SES (27.4%) (*p* < 0.001). All linear trends were statistically significant (all Ps < 0.001). More respondents with high SES also reported benefits on family mental health mainly due to increased family happiness (*p* for trend = 0.005) and fewer reported decreased family happiness (*p* for trend = 0.013). Significant trends were also observed for increased (benefit) and decreased (harm) family’s ability to cope with difficulty (*p* for trend = 0.006 and 0.032, respectively). We also analyzed the differences in perceived benefits and harms by education, income, housing, and number of cohabitants (Supplementary Table S1). The results were consistent with those by SES scores. More respondents with higher education reported perceived benefits on family physical and mental health and family relationships than those less educated. Moreover, more respondents with more income reported perceived benefits on family mental health and family relationships. More respondents with owned housing reported more perceived benefit on family physical health but it was marginally significant. On the other hand, fewer respondents with higher education, more income, and owned housing reported perceived harm of decreased family income than those less educated. 

### 3.3. Adjusted Odds Ratio of Perceived Benefits and Harms of COVID-19 for Sociodemographic Factors

Table 4 shows increased adjusted odds ratios (95% confidence interval) of perceived benefits on family mental health and relationships in females (1.44 (1.13–1.82), *p* = 0.003 and 1.44 (1.21–1.72), *p* < 0.001, respectively) and those aged 65 years or older (2.40 (1.17–4.92), *p* = 0.017 and 1.87 (1.06–3.31), *p* = 0.031, respectively). Middle and high SES showed increased ORs of perceived benefits on family physical health (1.49 (1.19–1.87), *p* = 0.001 and 1.57 (1.26, 1.96), *p* < 0.001, respectively), mental health (1.72 (1.19–2.48), *p* = 0.004 and 1.76 (1.23–2.51), *p* = 0.002, respectively), and relationships (1.71 (1.30–2.23), *p* < 0.001 and 1.89 (1.45–2.45), *p* < 0.001, respectively) compared with low SES.

Table 5 shows decreased adjusted ORs (95% confidence interval) of perceived harms on family mental health in females (0.81 (0.71–0.93), *p* = 0.002). Decreased ORs of perceived harms on family relationships (0.24 (0.13–0.46), *p* < 0.001) and income (0.52 (0.32–0.85), *p* = 0.008) were observed in those aged 65 years or older. Middle and high SES showed decreased ORs of perceived harms on family relationships (0.76 (0.61–0.96), *p* = 0.020 and 0.80 (0.64–0.99), *p* = 0.041, respectively) and family income (0.69 (0.57–0.83), *p* < 0.001 and 0.53 (0.45–0.64), *p* < 0.001, respectively).

## 4. Discussion

Our study is the first to show both perceived harms and benefits of the COVID-19 pandemic on several aspects of family well-being, such as family physical and mental health, family relationships, and income in the general population, and sociodemographic disparities of benefits and harms. Our results on benefits have extended the qualitative results on family relationships in the Spanish survey [19]. During the COVID-19 pandemic, staying at home increased the time spent with families, which increased mental health problems [27] and conflicts but may have also developed closer relationships [28]. Moreover, our results on the perceived benefits and harms of COVID-19 on family well-being more broadly included family physical and mental health, and family relationships and income. We have also first shown higher prevalence of harms of up to 37.9% on family mental health and 37.8% on family income but lower prevalence of benefits on family physical health (19.0%). Harms on family relationships were more than benefits (18.6% vs. 13.5%). Although no other studies were available for comparison, the prevalence estimates of both perceived harms and benefits were not strikingly high, probably because the first two COVID-19 waves in Hong Kong were successfully controlled with voluntary mass masking and social distancing but no lockdown. As the pandemic continued with the more serious third wave (July–August 2020) and the present fourth wave (from November 2020), more harms would emerge while benefits would become uncertain. Further surveys are needed to assess the dynamics of harms and benefits at different stages of the pandemic. 

Our results also showed socioeconomic disparity of perceived benefits and harms of COVID-19 on family well-being. Consistent with results from mainland China, which showed those with higher education and income were less likely to have income loss [18], our study showed that fewer respondents with high SES reported decreased family income. This may be explained by that less educated people or those with less income may be more likely to work in sectors that were affected most by social distancing measures (e.g., retail and catering). Moreover, more respondents with low SES reported perceived harms on family relationships and fewer reported benefits on family physical and mental health, and family relationships, suggesting that the COVID-19 pandemic leads to greater negative influences on underprivileged families in all aspects of family well-being in addition to family income. Financial insecurity is a major family stressor that may lead to a tense family environment and family conflicts [28,29,30]. More financial assistance to families in need would reduce not only financial hardships but probably also reduce socioeconomic disparity from the impact of the COVID-19 pandemic.

Despite the lower prevalence of benefits than harms, for family physical and mental health, high SES showed significantly increased adjusted OR of benefits but the decreased adjusted OR of harms were marginally significant. These suggest that such socioeconomic disparities were more remarkable for getting benefits than reducing harms. While the risks of harms did not differ substantially by SES, those with higher SES would be more likely to benefit from the pandemic. Existing studies showed that those with less education or less income were more likely to lose their job due to the pandemic [21]. Financial loss and inadequate resources were reported as major psychological stressors [11,31], suggesting that those with less resources tended to be more affected by the pandemic. Moreover, those with less income or education were more likely to be those who need to work despite the higher risk from unavoidable contact with many others [21]. Fear of being infected could be another psychological stressor [31]. On the other hand, those with higher income or education were more likely to work in sectors where flexible work arrangements (such as work from home) were feasible [21]. A qualitative analysis suggested that staying at home may result in improvement of family relationships, such as family (re)connection and acknowledgment, better communication, and emotional expressiveness [19]. We did not collect information on work arrangements and employment status and could not explore whether flexible work arrangements and employment status could explain the SES disparities on perceived benefits of COVID-19. However, our results have extended the existing results by showing that those with high SES (i.e., with more resources) were not only less affected but also reported more benefits from the pandemic [11].

Unexpectedly, more of our female respondents reported benefits on family mental health and relationships but more males reported harms on family mental health despite the prevalence estimates of perceived benefits on family mental health were rather low (9.0% and 7.0%, respectively). Gendered division of paid and unpaid work (housework) had been imbalanced, and women were more dissatisfied with work–life balance before the COVID-19 pandemic [17,32]. This gap could have been narrowed during the COVID-19 pandemic. Working at home as one of the key social distancing measures brought paid work home, forcing men to share more household work with women than pre-outbreak [32]. Compared with women, many more men reported negative feeling on the arrangement of paid and unpaid work and time allocation, which could narrow the gendered gap of dissatisfaction on work–life balance, during the outbreak [17]. This suggested that working women may benefit more from the pandemic than men. However, families with young children remained more likely to rely on women for childcare; in these families, women were more likely to bear excess childcare due to closure of childcare centers [16]. On the other hand, as some women were housewives, they were less directly affected by job loss, no pay leave, or work from home due to COVID-19 than men. In this case, women might have played more constructive roles in maintaining family well-being than men during this period. Sex differences in perceived benefits and harms of COVID-19 may be moderated by employment status, family composition, marital status, and family childcare. However, such information was not collected, and we could not explore differences among housewives and working women and men in families with versus without young children. More research on sex differences in family roles and their association with family functioning is warranted.

Older people are most vulnerable to COVID-19 and would be expected to be more distressed. However, our study showed that fewer older people aged 65 years or above reported perceived harms but more reported perceived benefits of the COVID-19 pandemic than younger people. This could be explained by young people having more problems and stress from uncertainties in studies and employment due to COVID-19 than older and retired people. Moreover, Hong Kong had few deaths from COVID-19 in the first two waves and older people who experienced SARS in 2003 would consider COVID-19 less harmful and fatal.

Our study provided some results of potential impact of COVID-19 on family well-being and related sociodemographic disparities. Sociodemographic factors were associated with risk perception, fear of being stigmatized or discriminated against, and vaccine hesitancy, that in turn, may influence health behaviours as well as mental health [33,34,35,36,37]. However, we did not include such questions because the questionnaire was already very long. Future studies should examine the associations of such factors with SES and perceived harms and benefits of COVID-19.

Methodologically, our study had the strength in using two simple questions on perceived harms and benefits of the COVID-19 pandemic, each with multiple options. Although these questions could minimize the respondents’ burden, we only got “yes/no” answers. Further research to obtain more details and explore the mechanisms are warranted. Our study had some limitations. All data were self-reported. The cross-sectional design could only show associations. However, the present analysis aimed to show sociodemographic disparities, not causation. Another limitation was the low participation rate. Under the dynamic and uncertain changes in the COVID-19 pandemic, inviting both probability- and non-probability-based online panels allowed us to collect a large sample within 6 days to maximize the statistical power and enhance the internal validity of cross-sectional (snapshot) results. In our literature search, no studies had collected as many as 5000 responses within 6 days. The overall prevalence was weighted by sex, age, and education of Hong Kong, which is an established method to address these potential biases [38]. The prevalence, even after weighting, might not be generalizable to the general population. However, the small differences of a few percentage points between the weighted and unweighted prevalence values suggested that the biases were not substantial. Moreover, the observed associations should be less affected. COVID-19 cases or people who had infected relatives might report more perceived harms and less benefits than those not affected. Our study did not have such information. However, less than one respondent was expected to be a confirmed COVID-19 case in our sample because only about 1100 (0.017% of our target population of about 6.5 million) confirmed cases were reported during our survey period. Our results may not be generalizable to other cities in China or other countries without lockdown because the responses to the COVID-19 pandemic in Hong Kong (e.g., no lockdown and 100% voluntary mask wearing) and socioeconomic impact of COVID-19 were different from other cities in China or other countries.

## 5. Conclusions

Our results of two simple questions showed both perceived harms and benefits of the COVID-19 pandemic on family well-being in all aspects even after the first two waves of COVID-19 were under control. Sociodemographic disparities were more remarkable for perceived benefits than harms of COVID-19. As the pandemic continues with uncertainties, further studies on the dynamic of harms and benefits are needed. Policymakers should provide urgent and additional assistance to underprivileged families and at-risk individuals to reduce inequities and support studies to monitor the impacts of the pandemic, including perceived benefits and harms, and examine the causal, mediating, and modifying factors.

## Figures and Tables

**Table 1:** Sociodemographic characteristics and perceived benefits and harms of the sample.

	Unweighted *n* (%)	Weighted *n* (%)
Sociodemographic characteristics		
Sex		
Males	2138 (43.7)	2295 (47.1)
Females	2753 (56.3)	2583 (52.9)
Age group, years		
18–24	219 (4.5)	416 (8.5)
25–44	2449 (50.1)	1581 (32.4)
45–64	2013 (41.2)	1839 (37.7)
65 or above	210 (4.3)	1041 (21.3)
Education		
Secondary or below	659 (13.6)	3183 (65.7)
Postsecondary	4199 (86.4)	1662 (34.3)
Household monthly income per person		
Lower	1270 (29.8)	2201 (52.6)
Higher	2986 (70.2)	1986 (47.4)
Housing		
Rent	1603 (33.9)	1744 (36.6)
Owned	3120 (66.1)	3025 (63.4)
Socioeconomic score		
Low	790 (18.9)	2160 (52.3)
Middle	1497 (35.8)	1376 (33.3)
High	1891 (45.3)	595 (14.4)
Perceived benefits		
Family physical health (any of the following)	1036 (22.7)	873 (19.0)
Improved family hygiene	934 (20.4)	820 (17.8)
Improved family physical health	605 (13.3)	499 (10.9)
Family mental health (any of the following)	370 (8.2)	330 (7.2)
Decreased family negative emotion	80 (1.8)	94 (2.1)
Increased family positive emotion	192 (4.2)	234 (5.1)
Increased family happiness	240 (5.3)	184 (4.0)
Family relationship (any of the following)	750 (16.5)	619 (13.5)
Increased family harmony	403 (8.8)	363 (7.9)
Increased family’s ability to cope with difficulties	556 (12.2)	486 (10.6)
Perceived harms		
Poorer family physical health	129 (2.9)	104 (2.3)
Decreased family income	1447 (32.6)	1671 (37.8)
Family mental health (any of the following)	1626 (36.9)	1669 (37.9)
Increased family negative emotion	1437 (32.6)	1465 (33.3)
Decreased family happiness	738 (16.7)	837 (18.9)
Family relationship (any of the following)	864 (19.6)	819 (18.6)
Decreased family harmony	508 (11.5)	521 (11.8)
Decreased family’s ability to cope with difficulties	113 (2.5)	101 (2.3)
Increased family conflicts	677 (15.3)	594 (13.4)

Missing data were excluded. Data were weighted by sex, age, and education of the 2019 Hong Kong population. Socioeconomic score: a composite score of education, household monthly income per person, and housing analyzed as low (0–1), middle (2), and high (3).

**Table 2:** Perceived benefits and harms of COVID-19 by sex and age.

	Sex			Age (Years)					
	Males	Females		18–24	25–44	45–64	65 or Above		
	*n* (%)	*n* (%)	*p* value	*n* (%)	*n* (%)	*n* (%)	*n* (%)	*p* value	*p* value for trend
Perceived benefits									
Family physical health (any of the following)	412 (20.8)	624 (24.2)	0.006	46 (23.0)	528 (23.2)	407 (21.5)	55 (27.9)	0.19	0.93
Improved family hygiene	374 (18.8)	560 (21.7)	0.02	45 (22.5)	481 (21.1)	355 (18.8)	53 (26.9)	0.024	0.57
Improved family physical health	251 (12.6)	354 (13.7)	0.29	28 (14.0)	279 (12.2)	262 (13.9)	36 (18.3)	0.069	0.04
Family mental health (any of the following)	138 (7.0)	232 (9.0)	0.01	16 (8.0)	152 (6.7)	173 (9.2)	29 (14.7)	<0.001	<0.001
Decreased family negative emotion	35 (1.8)	45 (1.8)	0.96	4 (2.0)	28 (1.2)	43 (2.3)	5 (2.5)	0.057	0.03
Increased family positive emotion	75 (3.8)	117 (4.6)	0.21	9 (4.5)	54 (2.4)	106 (5.7)	23 (11.9)	<0.001	<0.001
Increased family happiness	91 (4.6)	149 (5.8)	0.07	10 (5.0)	115 (5.1)	103 (5.5)	12 (6.1)	0.89	0.44
Family relationship (any of the following)	281 (14.2)	469 (18.2)	<0.001	32 (16.0)	328 (14.4)	345 (18.3)	45 (23.1)	0.001	<0.001
Increased family harmony	154 (7.8)	249 (9.7)	0.02	16 (8.0)	162 (7.1)	194 (10.3)	31 (15.9)	<0.001	<0.001
Increased family’s ability to cope with difficulties	213 (10.7)	343 (13.3)	0.008	23 (11.5)	230 (10.1)	267 (14.1)	36 (18.3)	<0.001	<0.001
Perceived harms									
Poorer family physical health	57 (3.0)	72 (2.9)	0.85	10 (5.0)	60 (2.7)	56 (3.1)	3 (1.6)	0.20	0.38
Decreased family income	650 (33.7)	797 (31.8)	0.17	75 (37.3)	725 (32.8)	598 (32.6)	49 (25.9)	0.12	0.09
Family mental health (any of the following)	750 (39.3)	876 (35.1)	0.005	65 (32.3)	828 (37.7)	672 (37.0)	61 (32.3)	0.25	0.76
Increased family negative emotion	659 (34.4)	778 (31.1)	0.02	60 (29.9)	733 (33.2)	588 (32.4)	56 (29.6)	0.59	0.64
Decreased family happiness	362 (18.9)	376 (15.0)	0.001	31 (15.4)	388 (17.6)	287 (15.7)	32 (16.9)	0.43	0.37
Family relationship (any of the following)	343 (17.9)	521 (20.9)	0.013	57 (28.4)	522 (23.7)	267 (14.6)	18 (9.6)	<0.001	<0.001
Decreased family harmony	236 (12.3)	272 (10.9)	0.15	35 (17.4)	307 (13.9)	157 (8.6)	9 (4.8)	<0.001	<0.001
Decreased family’s ability to cope with difficulties	52 (2.7)	61 (2.4)	0.57	10 (5.0)	53 (2.4)	43 (2.6)	2 (1.1)	0.082	0.18
Increased family conflicts	250 (13.0)	427 (17.0)	<0.001	47 (23.4)	424 (19.2)	193 (10.5)	13 (6.9)	<0.001	<0.001

Missing data were excluded. Data were unweighted.

**Table 3:** Perceived benefits and harms of COVID-19 by socioeconomic score.

	Socioeconomic Score				
	Low (0–1)	Middle (2)	High (3)		
	*n* (%)	*n* (%)	*n* (%)	*p* value	*p* value for trend
Perceived benefits					
Family physical health (any of the following)	129 (17.5)	334 (23.7)	434 (24.4)	0.001	0.001
Improved family hygiene	121 (16.3)	298 (21.1)	390 (21.9)	0.005	0.004
Improved family physical health	76 (10.3)	183 (13.0)	264 (14.8)	0.008	0.002
Family mental health (any of the following)	41 (5.6)	125 (8.9)	156 (8.8)	0.014	0.021
Decreased family negative emotion	13 (1.8)	24 (1.7)	29 (1.6)	0.97	0.81
Increased family positive emotion	28 (3.8)	59 (4.2)	81 (4.6)	0.67	0.37
Increased family happiness	21 (2.8)	80 (5.7)	105 (5.9)	0.005	0.005
Family relationship (any of the following)	83 (11.3)	244 (17.4)	330 (18.6)	<0.001	<0.001
Increased family harmony	39 (5.3)	137 (9.7)	176 (9.9)	0.001	0.001
Increased family’s ability to cope with difficulties	71 (9.6)	173 (12.3)	242 (13.6)	0.02	0.006
Perceived harms					
Poorer family physical health	25 (3.5)	39 (2.8)	40 (2.3)	0.24	0.091
Decreased family income	294 (41.0)	447 (32.5)	476 (27.4)	<0.001	<0.001
Family mental health (any of the following)	270 (38.0)	498 (36.4)	614 (35.6)	0.54	0.28
Increased family negative emotion	241 (33.9)	441 (32.2)	533 (30.9)	0.33	0.14
Decreased family happiness	135 (18.9)	226 (16.4)	257 (14.8)	0.04	0.01
Family relationship (any of the following)	159 (22.3)	248 (18.1)	324 (18.7)	0.051	0.096
Decreased family harmony	93 (13.0)	150 (10.9)	174 (10.0)	0.10	0.04
Decreased family’s ability to cope with difficulties	21 (2.9)	40 (2.9)	30 (1.7)	0.06	0.03
Increased family conflicts	121 (16.9)	191 (13.9)	264 (15.2)	0.18	0.53

Missing data were excluded. Data were unweighted. Socioeconomic score: a composite score of education, household monthly income per person, and housing analyzed as low (0–1), middle (2), and high (3).

**Table 4:** Association of perceived benefits of COVID-19 on family well-being with demographic and socioeconomic factors.

	Family Physical Health		Family Mental Health		Family Relationship	
	Adj. OR (95% CI)	*p* Value	Adj. OR (95% CI)	*p* Value	Adj. OR (95% CI)	*p* Value
Sex						
Males (ref)	1		1		1	
Females	1.25 (1.07, 1.45)	0.004	1.44 (1.13, 1.82)	0.003	1.44 (1.21, 1.72)	<0.001
Age group (years)						
18–24 (ref)	1	0.04 *	1	<0.001 *	1	<0.001 *
25–44	0.97 (0.65, 1.45)	0.90	0.83 (0.45, 1.54)	0.56	0.89 (0.56, 1.41)	0.61
45–64	0.87 (0.58, 1.30)	0.50	1.00 (0.54, 1.86)	0.99	1.14 (0.71, 1.81)	0.59
65 or above	1.45 (0.87, 2.40)	0.15	2.40 (1.17, 4.92)	0.02	1.87 (1.06, 3.31)	0.03
Socioeconomic score						
Low (ref)	1	<0.001 *	1	0.006 *	1	<0.001 *
Middle	1.49 (1.19, 1.87)	0.001	1.72 (1.19, 2.48)	0.004	1.71 (1.30, 2.23)	<0.001
High	1.57 (1.26, 1.96)	<0.001	1.76 (1.23, 2.51)	0.002	1.89 (1.45, 2.45)	<0.001

Sex, age group, and socioeconomic score were mutually adjusted. Data were unweighted. Socioeconomic score: a composite score of education, household monthly income per person, and housing analyzed as low (0–1), middle (2), and high (3). * *p* value for trend.

**Table 5:** Association of perceived harms of COVID-19 on family well-being with demographic and socioeconomic factors.

	Family Physical Health		Family Mental Health		Family Relationship		Family Income	
	Adj. OR (95% CI)	*p* Value	Adj. OR (95% CI)	*p* Value	Adj. OR (95% CI)	*p* Value	Adj. OR (95% CI)	*p* Value
Sex								
Males (ref)	1		1		1		1	
Females	0.89 (0.60, 1.32)	0.57	0.81 (0.71, 0.93)	0.002	1.07 (0.91, 1.26)	0.43	0.90 (0.78, 1.03)	0.13
Age group (years)								
18–24 (ref)	1	0.81 *	1	0.32 *	1	<0.001 *	1	0.04 *
25–44	0.85 (0.33, 2.17)	0.73	1.23 (0.86, 1.75)	0.26	0.74 (0.51, 1.06)	0.10	0.86 (0.61, 1.22)	0.40
45–64	0.92 (0.36, 2.36)	0.86	1.22 (0.85, 1.74)	0.28	0.41 (0.28, 0.60)	<0.001	0.84 (0.59, 1.19)	0.32
65 or above	0.53 (0.12, 2.27)	0.39	0.94 (0.59, 1.51)	0.80	0.24 (0.13, 0.46)	<0.001	0.52 (0.32, 0.85)	0.008
Socioeconomic score								
Low (ref)	1	0.23 *	1	0.38 *	1	0.053 *	1	<0.001 *
Middle	0.80 (0.48, 1.34)	0.39	0.91 (0.76, 1.10)	0.35	0.76 (0.61, 0.96)	0.02	0.69 (0.57, 0.83)	<0.001
High	0.64 (0.38, 1.07)	0.09	0.88 (0.73, 1.06)	0.17	0.80 (0.64, 0.99)	0.04	0.53 (0.45, 0.64)	<0.001

Sex, age group, and socioeconomic score were mutually adjusted. Data were unweighted. Socioeconomic score, a composite score of education, household monthly income per person, and housing analyzed as low (0–1), middle (2), and high (3) * *p* value for trend.

## Data Availability

The data presented in this study are available on request from the corresponding authors. The data are not publicly available because our analyses and paper writing on the results are in progress.

## Supplementary Materials

The following are available online at https://www.mdpi.com/1660-4601/18/3/1217/s1, Table S1: Perceived benefits and harms of COVID-19 by education, housing and family income.
