# Supplementary material for: Perceived Benefits and Harms of the COVID-19 Pandemic on Family Well-Being and Their Sociodemographic Disparities in Hong Kong: A Cross-Sectional Study
Source: Int J Environ Res Public Health. 2021 Jan 29;18(3):1217. doi: 10.3390/ijerph18031217 (PMC7908477; doi:10.3390/ijerph18031217)
Supplement: Supplementary file 1 [file ijerph-18-01217-s001.pdf]

International Journal of
Environmental Research and Public Health

MDPI

Supplementary 1. Perceived benefits and harms of COVID-19 by education, housing and family income

	**Education**			**Housing**			**Household income (by household size)**		
	**Secondary or lower**	**Post-secondary**		**Rent**	**Owned**		**<=Median HK household monthly income**	**>Median HK household monthly income**	
	n (%)	n (%)	P value	n (%)	n (%)	P value	n (%)	n (%)	P value
**Perceived benefits**									
**Family physical health (any of the following)**	**94 (15.1)**	**934 (23.9)**	**<0.001**	**317 (21.2)**	**687 (23.6)**	**0.08**	**249 (21.2)**	**660 (23.4)**	**0.12**
Improved family hygiene	89 (14.3)	837 (21.4)	<0.001	292 (19.5)	613 (21.0)	0.23	224 (19.0)	595 (21.1)	0.13
Improved family physical health	53 (8.5)	548 (14.0)	<0.001	178 (11.9)	407 (14.0)	0.06	142 (12.1)	389 (13.8)	0.14
**Family mental health (any of the following)**	**32 (5.2)**	**336 (8.6)**	**0.004**	**127 (8.5)**	**231 (8.0)**	**0.51**	**80 (6.8)**	**248 (8.9)**	**0.03**
Decreased family negative emotion	12 (1.9)	67 (1.7)	0.69	31 (2.1)	45 (1.6)	0.20	17 (1.5)	53 (1.9)	0.34
Increased family positive emotion	24 (3.9)	167 (4.3)	0.63	54 (3.6)	130 (4.5)	0.18	50 (4.3)	122 (4.4)	0.90
Increased family happiness	18 (2.9)	220 (5.6)	0.005	81 (5.4)	151 (5.2)	0.74	45 (3.8)	166 (5.9)	0.008
**Family relationship (any of the following)**	**65 (10.5)**	**682 (17.5)**	**<0.001**	**248 (16.6)**	**483 (16.6)**	**1.00**	**147 (12.6)**	**516 (18.4)**	**<0.001**
Increased family harmony	35 (5.6)	367 (9.4)	0.002	130 (8.7)	263 (9.0)	0.71	77 (6.6)	280 (10.0)	0.001
Increased family's ability to cope with difficulties	54 (8.7)	499 (12.7)	0.004	182 (12.1)	357 (12.2)	0.93	116 (9.8)	376 (13.3)	0.002
**Perceived harms**									
**Poorer family physical health**	**13 (2.2)**	**116 (3.0)**	**0.24**	**46 (3.2)**	**80 (2.8)**	**0.54**	**45 (3.9)**	**60 (2.2)**	**0.002**
**Family mental health (any of the following)**	**239 (40.2)**	**1377 (36.4)**	**0.070**	**536 (37.0)**	**1034 (36.7)**	**0.85**	**422 (37.1)**	**982 (35.9)**	**0.50**
Increased family negative emotion	208 (35.0)	1221 (32.2)	0.17	485 (33.4)	902 (32.0)	0.35	379 (33.3)	856 (31.3)	0.22
Decreased family happiness	118 (19.7)	614 (16.1)	0.03	256 (17.6)	458 (16.2)	0.24	203 (17.7)	424 (15.4)	0.07
**Family relationship (any of the following)**	**113 (18.9)**	**746 (19.6)**	**0.67**	**297 (20.4)**	**542 (19.2)**	**0.33**	**243 (21.3)**	**499 (18.2)**	**0.022**
Decreased family harmony	73 (12.2)	431 (11.3)	0.54	186 (12.8)	302 (10.7)	0.04	140 (12.3)	286 (10.4)	0.09
Decreased family's ability to cope with difficulties	15 (2.5)	98 (2.6)	0.92	51 (3.5)	58 (2.0)	0.004	32 (2.8)	59 (2.1)	0.22
Increased family conflicts	77 (12.9)	597 (15.7)	0.08	223 (15.3)	435 (15.3)	0.95	191 (16.7)	393 (14.3)	0.06
**Decreased family income**	**255 (42.6)**	**1183 (31.0)**	**<0.001**	**528 (36.2)**	**876 (30.9)**	**0.001**	**437 (38.1)**	**794 (28.9)**	**<0.001**

Missing data was excluded. Data was unweighted.
